# GM-CSF and IL-33 Orchestrate Polynucleation and Polyploidy of Resident Murine Alveolar Macrophages in a Murine Model of Allergic Asthma

**DOI:** 10.3390/ijms21207487

**Published:** 2020-10-11

**Authors:** Katharina M. Quell, Kuheli Dutta, Ülkü R. Korkmaz, Larissa Nogueira de Almeida, Tillman Vollbrandt, Peter König, Ian Lewkowich, George S. Deepe, Admar Verschoor, Jörg Köhl, Yves Laumonnier

**Affiliations:** 1 Institute for Systemic Inflammation Research, University of Lübeck, 23538 Lübeck, Germany; katharina.quell@web.de (K.M.Q.); kuheli.dutta14@gmail.com (K.D.); RabiaUelkue.Korkmaz@uksh.de (Ü.R.K.); LarissaNogueirade.Almeida@uksh.de (L.N.d.A.); joerg.koehl@uksh.de (J.K.); 2 Cell Analysis Core Facility, University of Lübeck, 23538 Lübeck, Germany; Tillman.Vollbrandt@uksh.de; 3 Institute of Anatomy, University of Lübeck, 23538 Lübeck, Germany; koenig@anat.uni-luebeck.de; 4 Airway Research Center North, Member of the German Center for Lung Research (DZL), 23538 Lübeck, Germany; 5 Division of Immunobiology, Cincinnati Children’s Hospital, Cincinnati, OH 45229, USA; Ian.Lewkowich@cchmc.org; 6 Department of Pediatrics, University of Cincinnati, Cincinnati, OH 45229, USA; 7 College of Medicine, University of Cincinnati, Cincinnati, OH 45229, USA; Deepegs@ucmail.uc.edu; 8 Department of Infectious Diseases and Microbiology, University of Lübeck, 23538 Lübeck, Germany; Admar.Verschoor@uksh.de

**Keywords:** allergic asthma, alveolar macrophages, division defect, polynucleation, mouse model

## Abstract

Allergic asthma is a chronical pulmonary disease with high prevalence. It manifests as a maladaptive immune response to common airborne allergens and is characterized by airway hyperresponsiveness, eosinophilia, type 2 cytokine-associated inflammation, and mucus overproduction. Alveolar macrophages (AMs), although contributing to lung homeostasis and tolerance to allergens at steady state, have attracted less attention compared to professional antigen-presenting and adaptive immune cells in their contributions. Using an acute model of house dust mite-driven allergic asthma in mice, we showed that a fraction of resident tissue-associated AMs, while polarizing to the alternatively activated M2 phenotype, exhibited signs of polynucleation and polyploidy. Mechanistically, in vitro assays showed that only Granulocyte-Macrophage Colony Stimulating Factor and interleukins IL-13 and IL-33, but not IL-4 or IL-5, participate in the establishment of this phenotype, which resulted from division defects and not cell-cell fusion as shown by microscopy. Intriguingly, mRNA analysis of AMs isolated from allergic asthmatic lungs failed to show changes in the expression of genes involved in DNA damage control except for *MafB*. Altogether, our data support the idea that upon allergic inflammation, AMs undergo DNA damage-induced stresses, which may provide new unconventional therapeutical approaches to treat allergic asthma.

## 1. Introduction

Asthma is a widespread disease affecting 300 million people around the world and estimations predict that 100 million more will be affected by 2050 [1]. It is characterized by airway hyper-responsiveness, narrowing of the airways, inflammatory cell recruitment, and increased mucus production [2]. Further, allergic asthma is characterized by an allergen-triggered eosinophilic infiltration; the presence of type 2 cytokines such as interleukin (IL)-4, IL-5, and IL-13; and the presence of Immunoglobulin IgE [2]. By suppressing inflammatory responses through the secretion of anti-inflammatory cytokines such as IL-10 and IL-12, Alveolar macrophages (AMs) are associated, at steady state, with the maintenance of immunological homeostasis of the lungs [3]. During the sensitization phase, resident AMs maintain homeostasis, until high levels of cytokines such as IL-4, IL-13, and interferon (IFN)-γ are reached and the phenotype of AMs changes to a more pro-inflammatory one [3]. In addition, macrophages derived from peripheral blood-recruited monocytes infiltrate the lungs where they secrete additional pro-inflammatory mediators [3]. However, as the inflammatory response matures, resident AMs develop an anti-inflammatory phenotype, which ultimately tames and resolves the inflammation and assists in tissue remodeling [4].

Interestingly, one unique feature of macrophages is their ability to form multinucleated giant cells (MNGCs). Although their functions remain elusive, these MNGCs are thought to be responsible for engulfing large particles like foreign material, intracellular bacteria, parasites, and fungi [5]. In addition, they participate in physiological processes including the formation of osteoclasts, which resorb bone tissue; or the formation of foreign body giant cells, which degrade and resorb foreign materials [6]; and promote tissue repair and remodeling [7]. However, while these functions are often considered anti-inflammatory, MNGCs are also found in pathophysiological conditions, in particular in granuloma-associated diseases [5] and promote the progression/resolution of various diseases [8].

To date, multinuclear or polynuclear AMs have solely been identified in bronchoalveolar lavage of patients with asbestos exposure-induced interstitial lung disease [9,10], while polynucleation has been reported in a murine model of allergic asthma [11]. Although MNGCs in bones form in response to Th2-related inflammation, in particular upon IL-4 exposure [12,13], no mechanism has been proposed to explain the formation of MNGCs or polynucleated macrophages in allergic inflamed lungs. Currently, MNGCs arise either from cell-cell fusion mechanisms [6] or DNA damage and division defects in tuberculosis (TB)-associated granuloma [14].

Here we show that following the induction of allergic lung inflammation, driven by repeated exposure to house dust mite extract (HDM), a fraction of the MHCII^+^ alveolar macrophages (AMs) exhibits a polynuclear phenotype. These macrophages can be identified by flow cytometric analysis from lung cell isolates and by histology. Furthermore, in vitro studies demonstrate that activation of steady state primary AMs with growth factor such as Granulocyte Macrophage-Colony Stimulating Factor (GM-CSF), and cytokines such as IL-13 and IL-33, but not IL-4 or IL-5, are instrumental to drive polynucleation and polyploidy of AMs. Further, in vitro assays, mRNA analysis, and visual examination of polynuclear macrophages suggest that they arise principally from cell division defects rather than cell-cell fusion.

## 2. Results

### 2.1. Allergic Asthma-Driven Inflammatory Conditions Trigger an M2 Activation of Alveolar Macrophages

**Figure 1:**
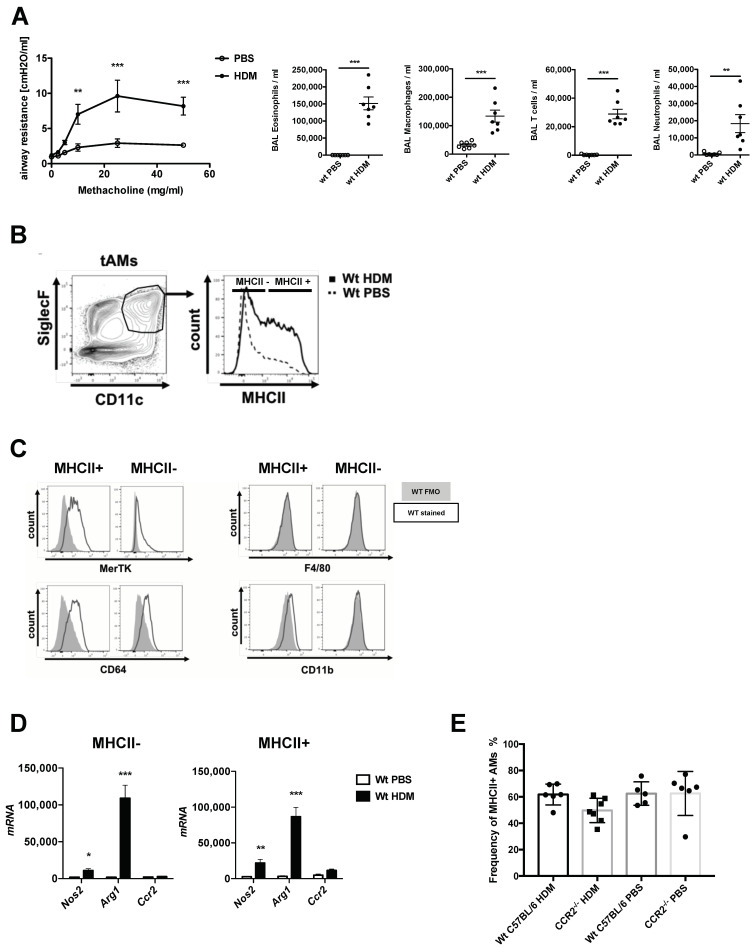
Allergic asthmatic inflammatory conditions drive a heterogenous expression of MHCII in tissue resident alveolar macrophages. (**A**) Characterization of the allergic asthma phenotype of mice exposed to house dust mite extract (HDM) compared to non-asthmatic control (PBS). Upon HDM exposure, mice show increased airway hyperresponsiveness (AHR) upon methacoline exposure, as shown by increased airway resistance and strong inflammatory cell influx consisting mostly in eosinophils, T cells and to a lower extent neutrophils. Data show mean value ± SEM; *n* = 7–8 per group. Statistical significance in the AHR dose response curves was assessed using a two ways ANOVA. Statistical significance of the cellular recruitments in the airways was assessed using a *t*-test. ** *p* < 0.01; *** *p* < 0.001. (**B**) CD11c^+^SiglecF^+^ resident tissue-associated alveolar macrophages (tAMs) express different levels of MHCII complex upon HDM-driven allergic asthma inflammation. (**C**) Flow cytometric assessment of different markers of MHCII+ and MHCII− tAMs of HDM-treated mice, based on the gating provided in (B). Histograms show FMO control (grey) and MHCII signal (black) in the two subpopulations. The markers tested at the surface of CD11c^+^SiglecF^+^ tAMs were MerTK, CD64 and classical macrophages surface markers such as CD11b and F4/80. (**D**) RT-PCR analysis of MHCII+ and MCHII− tAMs for mRNA levels of *Nos2*, *Arg1* and *Ccr2* upon allergic inflammation. Data show mean value ± SEM of mRNA abundance reported to *S14* mRNA in sorted MHCII+ and MCHII− tAMs; *n* = 4–7 per group. Statistical significance between PBS and HDM samples was assessed using a *t*-test; * *p* < 0.05; ** *p* < 0.01; *** *p* < 0.001. (**E**) Frequency of MHCII+ AMs in lungs of C57BL/6 WT and *Ccr2*^−/−^ mice upon HDM-driven allergic asthma. Data show mean value ± SEM; *n* = 5–6 animals.

Activation of alveolar macrophages is associated with allergic asthma-driven inflammation. Intratracheal (HDM) exposure induced (Figure S1A) robust airway hyperresponsiveness (AHR) and strong inflammation of the airways, characterized mainly by an infiltration of eosinophils and T cells (Figure 1A) in BALB/c mice. Furthermore, SiglecF^+^CD11c^+^ tissue-associated alveolar macrophages (tAMs) showed an upregulation of MHCII expression in HDM-treated mice, compared to the PBS-treated control, 72 h after the final HDM exposure (Figure 1B). This led to a heterogeneous population of tAMs characterized by high and low MHCII expression. To further evaluate the characteristics of MHCII^+^ versus MHCII^-^ populations, we examined the expression of additional specific macrophage markers. In both HDM-triggered MHCII^+^ and MHCII^−^ cells, we observed similar levels of MerTK and CD64 (Figure 1C), while they remained low for CD11b and negative for F4/80 (Figure 1C), confirming their resident AM phenotype. Moreover, RT-PCR analysis of FACS-purified MHCII^+^ and MHC^-^ populations from both PBS- and HDM-exposed animals demonstrated that HDM-exposure was uniquely associated with a shift towards an M2 macrophage phenotype characterized by high levels of *Arg1* and low levels of *Nos2* (Figure 1D). Both MHCII^+^ and MHCII^-^ populations demonstrated similar skewing (Figure 1D). Further, both MHCII^+^ and MHCII^−^ AMs showed a low expression level of *Ccr2* mRNA (Figure 1D), and HDM sensitization of *Ccr2*^−/−^ mice did not result in changes in the frequency of MHCII^+^ tAMs (Figure 1E), suggesting that they did not derive from blood-recruited monocytes.

### 2.2. HDM-Driven MHCII^+^ Activated tAMs Exhibit Signs of Polynucleation

**Figure 2:**
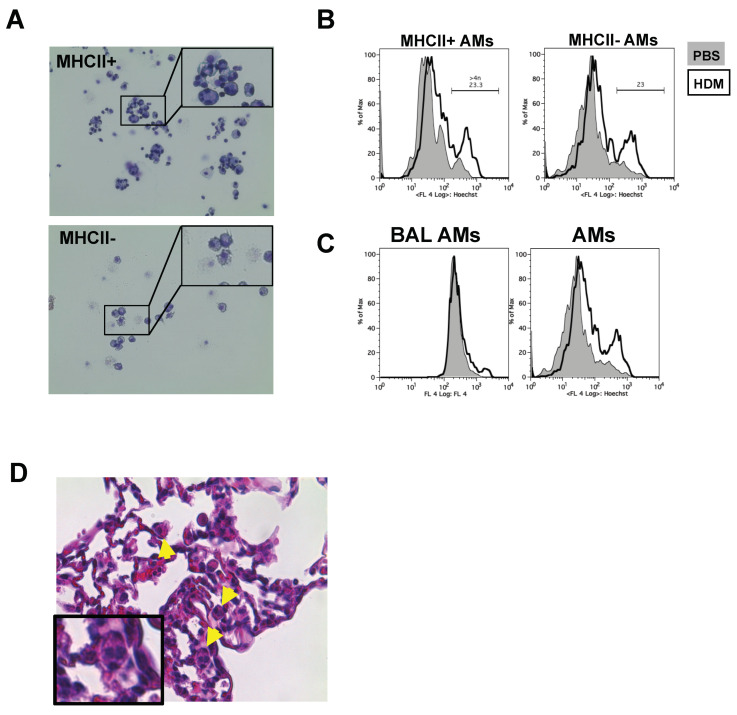
MHCII^+^ tAMs subpopulation shows signs of polynucleation upon allergic asthma (**A**) CD11c^+^SiglecF^+^MHCII^+^ resident tAMs from HDM-triggered asthmatic lungs but not the MCHII- subpopulation; showing cells with four to six nuclei. Data shown are representative of at least three independent experiments. Pictures were taken with a 20× objective. (**B**) CD11c^+^ SiglecF^+^ tAMs from HDM-induced asthma show increased DNA content by Hoechst 33342 staining. Data are representative of at least two independent experiments. (**C**) DNA content increase is observed in tissue tAMs but not in bronchoalveolar macrophages as shown by Hoechst 33342 staining. Data are representative of at least two independent experiments. (**D**) Histology section of HDM-treated mouse. Arrows indicate the presence of polynucleated cells in the alveolar compartment. Pictures were taken using a 20× objective.

We stained and sorted MHCII^−^ and MHCII^+^ tAMs from allergic inflamed lung tissue and examined their morphological appearance by May-Giemsa staining. As expected, MHCII^−^ tAMs showed characteristic macrophage morphology (Figure 2A). In contrast, examination of MHCII^+^ tAMs revealed that, in addition to such morphologically classical cells, several macrophages harbored more than two nuclei. However, these cells did not show distinct multinuclear and large expansion of the cell body, which is typical for large giant cells [15] (Figure 2A). These data suggested that upon induction of allergic inflammation, tAMs might form polynucleated cells although not MNGCs. Staining of DNA with Hoechst 33342 in both MHCII^−^ and MHCII^+^ populations showed an increase in the amount of DNA in HDM-treated cells compared to the PBS (Figure 2C), suggesting either polynucleation or polyploidy was present in HDM-treated tAMs. Of note, while polynuclear cells with high DNA content were observed in lung tissue preparations, we could observe neither polynucleated (data not shown) nor tAMs with high DNA content (polypoid) in bronchoalveolar lavages of allergic asthmatic mice (Figure 2C). To ensure that the polynucleated tAMs identified did not arise from an artifact related to lung digestion, isolation procedure and/or FACS sorting, we examined 50 μm lung histology sections of HDM-treated WT stained with H&E staining. Interestingly, we identified two to four nuclei polynucleated cells, located around the alveoli (Figure 2D). Altogether, these findings suggested that polyploid and polynuclear cells might develop, in vivo, from SiglecF^+^CD11c^+^ tAMs during the effector phase of allergic asthma.

### 2.3. In Vitro, GM-CSF Drives tAMs Polynucleation While IL-13 and IL-33 Polyploidy

**Figure 3:**
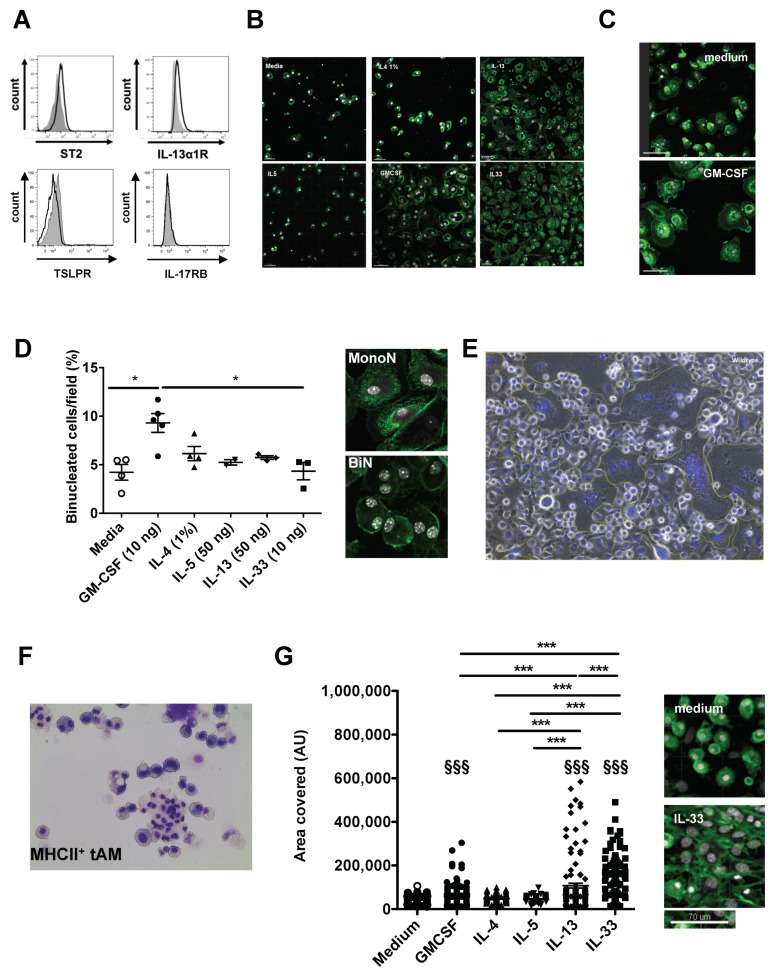
Tissue resident macrophages undergo binucleation upon GM-CSF treatment in vitro. (**A**) Resident tAMs express a receptor for IL-33 (ST2) and IL-13 (IL-13Ra), but not for IL-25 (IL-17RB) and TSLP as shown by flow cytometry. Histograms show signal (black line) compared to Fluorescence minus one (FMO) controls (grey histogram). Data are representative of at least two independent stainings. (**B**) Visual examination of cultures of sorted Siglec F^+^ CD11c^+^ tAMs after 7 days of culture with medium alone, IL-4 (1%), IL-13 (50 ng/mL), IL-5 (50 ng/mL), IL-33 (50 ng/mL) or GM-CSF (10 ng/mL). Slides were stained with FITC-wheat germ agglutinin (green) and counterstained with DAPI (white). Data are representative of at least three independent experiments. Pictures were taken using a ×20 oil-immersion objective. (**C**) Close-up view of cells cultivated in medium alone or upon GM-CSF. Cells stimulated with GM-CSF exhibited dendrites and better adherence compared to cells in medium alone. Pictures were taken using a ×40 objective. (**D**) Evaluation of the frequency of polynucleated macrophages upon various stimulii. Data are mean value ± SEM; *n* = 2–5. Pictures show a representative example of mono and binucleated cells observed with a 40× objective, * *p* < 0.05. (**E**) BM-derived macrophage fusion induced by the presence of 1% IL-4 conditioned medium in the cell culture medium. Cells were stained with Hemacolor and the picture was taken in bright field microscopy using a ×20 objective. Data are representative of two experiments. (**F**) CD11c^+^SiglecF^+^ MHCII^+^ resident tAMs from IL-33-triggered asthmatic lungs show cells with four to six nuclei after sorting, cytospinning and staining with diff-quick reagent. Data shown are representative of at least three independent experiments. Pictures were taken using a ×20 objective. (**G**) Area covered by the nuclei of tAMs cultured 7 days with the above-mentioned cytokines and growth factor (media change with cytokines at day 2, 3 and 5). The areas were measured after staining and microscopic evaluation, using Image J software. Pictures show a representative example of medium-treated and IL-33-treated cells using a 40× objective. Data are mean values ± SEM. Statistical significance was assessed using ANOVA. Asterisk refers to significance between the treatments, while § refers to significance compared to the medium control; ^§§§^ and *** *p* < 0.001.

To delineate the molecular mechanisms involved in the formation of polynucleated cells and to confirm their resident alveolar macrophage origin, we developed an in vitro assay to test the ability of type 2 cytokines and alarmin to induce tAM to polynuclear transformation. In addition to the reported expression of receptors for GM-CSF [16], IL-4 [17] and IL-5 [18,19], we found that steady state macrophages expressed the receptor for IL-33 (ST2) and IL-13 (IL-13Rα), but not Thymic stromal lymphopoietin (TSLP) receptor or the IL-17RB protein, the latter being a components of the IL-25 receptor (Figure 3A). Therefore, naïve tAMs from steady state lungs were FACS sorted based on their expression of Siglec F and CD11c, and stimulated in vitro for 7 days either with type 2 cytokines (IL-4, IL-5, IL-13) or IL-33, a well-known alarmin released upon allergen exposure by damaged epithelial cells. Further, we used GM-CSF, a major regulator of AMs differentiation that, similar to IL-4, has been shown to trigger the formation of multinucleated giant cell (MNGC) [15,20,21]. After fixation and staining cells with 4′,6-diamidino-2-phenylindole (DAPI) and FITC-labeled wheat germ agglutinin, we observed striking differences in cell density between medium controls, IL-4 or IL-5 stimulation on the one hand, and in cells stimulated with GM-CSF, IL-33 and to a lesser extent IL-13 on the other hand (Figure 3B). In addition to higher proliferation rates, GM-CSF-, IL-13- or IL-33-stimulated cells showed a healthier phenotype, characterized by strong adhesion and spreading of cellular dendrites. In contrast, cells kept in the media control or stimulated with IL-4 and IL-5 exhibited a rounded phenotype (Figure 3C). In the presence of GM-CSF, we observed binucleated (BiN) cells with nuclei frequently arranged symmetrically (Figure 3B,D), and the frequency of BiN cells was significantly increased in GM-CSF-stimulated tAMs compared to all other conditions (Figure 3D). BiN cells did not harbor a fragmented nucleus, and thus, did not appear apoptotic (Figure S1D). Although IL-4 drove bone marrow (BM)-derived macrophages into multinucleation in vitro (Figure 3E), it did not induce BiN tAMs formation, and the frequency of polynuclear macrophages upon IL-4 was similar to medium alone (Figure 3D). Intriguingly, neither IL-13 nor IL-33 triggered a clear increase in polynucleation frequency in vitro, although IL-33 given intra-tracheally to mice promoted a clear polynucleation phenotype in MHCII^+^ tAMs (Figure 3F). However, both IL-13- and IL-33-stimulated cells harbored significantly enlarged nuclei compared to medium, IL-4-, and IL-5-stimulated cells, and to a lesser extent to GM-CSF treated cells (Figure 3G), suggesting that IL-13 or IL-33 stimulation may trigger tAMs polyploidy rather than poynucleation.

### 2.4. GM-CSF-Driven Polynucleation of tAMs Resulted from Division Defects Rather Than Cell-Cell Fusion

**Figure 4:**
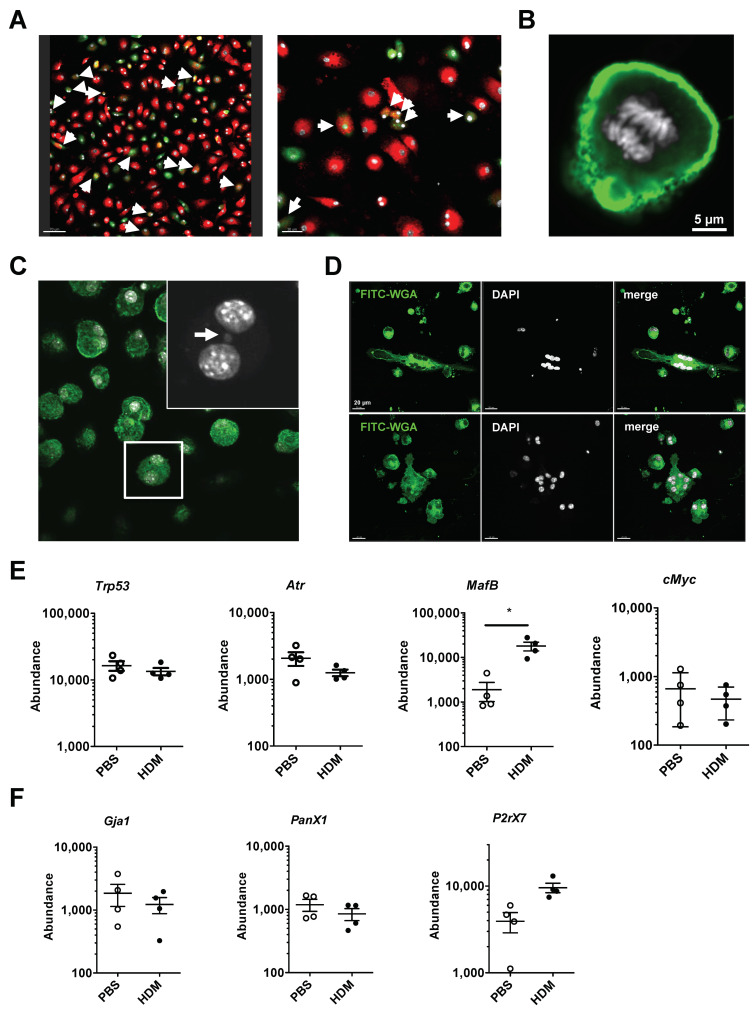
tAM polynucleation arise from division defect rather than cell fusion. (**A**) Assessment of fused macrophages by confocal microscopy (original magnification ×20/×40). Cell fusion was assayed by cultivating for 5 days with sorted SiglecF^+^ CD11c^+^ tAMs stained with PKH26 (red) or CFSE (green) and counterstained with DAPI (white). Bars represent 70/30 μm. Arrows show cells positive for both red and green staining. (**B**) SiglecF^+^ CD11c^+^ tAMs cultivated with GM-CSF show signs of proliferation. Cells were stained with FITC-WGA (green), counterstained with DAPI (white) and examined by fluorescence microscopy (original magnification ×60). (**C**) Evidences of division defect of tAMs by confocal microscopy (original magnification ×40). tAM cultivated with GM-CSF shows the presence of micronucleus (shown by an arrow). Cells were stained with FITC-WGA (green) and counterstained with DAPI (white). (**D**) tAMs sorted from four-steps HDM-triggered asthmatic lungs were kept for 24 h in presence of GM-CSF. Cells show symmetrical polynucleation. Cells were stained with FITC-WGA (green) and counterstained with DAPI (white). Data are representative of two independent experiments. Picture were taken using a ×40 objective. (**E**) Cells from PBS or HDM treated mice for 4 weeks were isolated and analyzed for RNA content. Data show abundance of *Trp53*, *Atr*, C-*myc,* and *Mafb* mRNA reported to *S14* level, in SiglecF^+^ CD11c^+^ MHCII+ tAMs. Values shown are the mean ± SEM from *n* = 4 isolations. Statistical significance was assessed using a t-test. * *p* < 0.05. (**F**) Expression level of *Gja1*, *P2rx7*, and *Panx1* in the same samples were evaluated. Data show abundance of mRNA reported to *S14* level, in MHCII+ tAMs. Values are the mean ± SEM from *n* = 4 isolations.

While the formation of polynuclear macrophages is typically thought to be driven by cellular fusion [20], recent data have indicated that polyploid granuloma-resident macrophages in *Mycobacterium Tuberculosis* (TB) infection are formed via altered cell division events and mitotic defects such as endoreplication and cytokinesis failure [14]. Therefore, we tested if the observed polynuclear cells, in our in vitro cultures, were generated by fusion or cell division defects. First, we purified naïve tAMs by FACS, labeled them either with PKH26 or carboxyfluorescein succinimidyl ester (CFSE) (Figure S1B) and combined the differently labeled tAMs in the presence of GM-CSF to induce BiN cell formation. After 5 days of co-culture, we identified BiN cells that were either PKH26^+^ or CFSE^+^, however only a few of them were PKH26^+^CFSE^+^ (Figure 4A). Although this may indicate that polynuclear macrophages were formed by fusion, we also observed mononucleated cells with a double positive signal, suggesting either phagocytosis of dying tAMs or cell fusion followed by nuclear fusion. However, careful examination of GM-CSF-treated tAMs in vitro cultures revealed that cells actively proliferated (Figure 4B), and micronuclei were observed (Figure 4C). Furthermore, tAMs present in the lung of allergic asthmatic mice exposed repeatedly to HDM over 4 weeks and cultured with GM-CSF for 24 h showed a symmetrical distribution of nuclei in the cells (Figure 4D), similar to polynuclear cells generated upon GM-CSF treatment of naive tAMs (Figure 3D). Altogether, our data suggested that cell division defects were the main mechanism involved in the formation of polynuclear tAMs.

### 2.5. HDM-Activated MHCII^+^ tAMs Express Higher Levels of MafB

Since in TB-associated granuloma formation, polynucleation and polyploidy are associated with alterations in DNA damage control and changes in expression of *Mafb, C-myc, Trp53,* and *Atr* [14], we assessed the mRNA expression of these genes in the MHCII^+^ subpopulations of HDM-triggered tAMs, by semi-quantitative real time PCR. Intriguingly, while the expression of *Mafb* was significantly increased in the HDM-treated mice in relation to the PBS treated mice, neither *Atr*, *Trp53* nor *C-myc* expression were altered upon HDM treatment in both the tAMs subpopulations compared to PBS controls (Figure 4E). However, in vitro, we observed that GM-CSF decreased the phosphorylation status of p53 in sorted AMs after 3 days of stimulation compared to non-stimulated controls (Supplementary Figure S2). In order to appreciate a contribution of cell fusion to the formation of polynuclear tAMs under allergic asthma conditions, we also delineated the expression of several genes associated to cell-cell fusion, such as *Gja1*, encoding Connexin 43, *P2x7* and *Panx1*. None of these genes increased in MHCII^+^ cells upon HDM compared to PBS controls (Figure 4F).

## 3. Discussion

Multinucleated or polynucleated giant cells are a common cell type encountered in various physiological and pathological situations, ranging from bone osteoclast differentiation to granuloma formations in tuberculosis (TB) [14,22], histoplasma [23], sarcoidosis [24], Takayasu and giant cell arteritis [25], and in airways of asbestos-exposed workers [10]. By contrast, evidence for their formation during allergic asthma is limited. Although some were identified in an OVA-driven allergic asthma model [11], polynucleation of macrophages has not been reported in fatal allergic asthmatic human lungs. Here, we show that upon allergen-induced lung inflammation in a mouse model, a significant fraction of tissue-associate alveolar macrophages (tAMs) underwent polynucleation and polyploidy, although we did not find either polynucleation or polyploidy in BAL alveolar macrophages from asthmatic animals. Mechanistically, GM-CSF was the main trigger for polynucleation while IL-33 and IL-13 were involved in polyploidy. Neither IL-4 nor IL-5 drove these processes. In addition, cell division defects were the main cause for the formation of polynucleated tAMs in vitro and in vivo.

We observed that not all cytokines present in the asthma-associated pro-inflammatory milieu were equal in driving polynucleation of tAMs. Indeed, while GM-CSF was a potent driver, IL-33 was not as active in vitro, although its in vivo effect was stronger. GM-CSF is a master regulator of AMs differentiation and functions [26,27] and participates, with IL-4, in the MNGC formation in vitro [20]. Interestingly, although we confirm IL-4-induced polynucleation of BM-derived macrophages [15], IL-4 did not induce polynucleation of tAMs. This apparent discrepancy may be linked to the very peculiar phenotype of AMs. Indeed, in contrast to “classical” macrophages, such as BM-derived macrophages differentiated either with Macrophage-Colony Stimulating Factor (M-CSF) or GM-CSF, peritoneal and visceral adipose tissue-associated AMs express neither F4/80 nor CD11b but Siglec F [28,29]. Further, unlike other macrophages, tAMs do not express the anaphylatoxin receptor C3aR [28], suggesting that the difference between AMs/tAMs and other macrophages is great in terms of functionality. Supporting the idea that different macrophages show different responses to IL-4, a recent study showed that F4/80^+^ macrophages respond differently to IL-4 trigger compared to CD68^+^ and CD11b^+^ macrophages [30]. GM-CSF is a crucial growth factor for the development and homeostasis of alveolar macrophages [29,31,32]. Interestingly, while M-CSF is a well-known trigger of monocyte/macrophage multinucleation by fusion [33], GM-CSF plays a more accessory role in these processes [21,34]. However, GM-CSF is secreted by TB mutinuclear cells in vitro [35] and may fuel the formation of polynuclear granuloma besides TLR-2 signaling [14]. Further, similar to our observations that GM-CSF drives alveolar macrophage proliferation, increased GM-SCF drives AMs accumulation in smokers [36]. Besides the release of GM-CSF, allergen exposure directly triggers the secretion of various alarmins such as IL-33, TSLP or IL-25 [37] by damaged epithelial cells, or upon HDM exposure, after transient release of GM-CSF itself [38]. However, while tAMs from naive mice express the receptor for GM-CSF [19], IL-13, and IL-33, we did not observe the expression of TSLPR or IL-17RB (the specific receptor chain involved in the formation of the IL-25 receptor) in steady state alveolar macrophages, therefore limiting our study to IL-13 and IL-33. Intriguingly, IL-13 has been reported to act as an inducer of fusion-driven MNGC formation [5] and contributes to macrophage polarization in sarcoidosis-associated granuloma formation in vitro [39]. However, in tAMs, our data suggest that IL-13 drives polyploidy. This could be the result of an alternative use of IL-13Ra2/STAT3 signaling rather IL-13Ra1/STAT6 in tAMs. Indeed, IL-13Ra2 has been shown to contribute to allergic asthma [40] and plays a unique role in the function of conventional pulmonary dendritic cells [41]. In contrast, although IL-33 influences polarization of alveolar macrophages [42], there is limited evidence for a role of IL-33 in the formation of giant cells, although it may be a decisive pro-inflammatory cytokine contributing to the pathology of Giant Cell Arteritis [43]. Conversely, granulomas have been reported to be a source of IL-33 in pulmonary sarcoidosis [44].

Mechanistically, we showed that division defect seems to be the main reason for the polynucleation of tAMs rather than cell fusion. In particular, we observed a symmetrical distribution of nuclei in cells as well as the presence of features such as micronuclei. Both result from migration delays of the chromatids [45] and occur singly or in a recurrent manner [14]. Interestingly, the presence of micronuclei has been shown to activate STING and cGAS pathways [46] and may be a cause for type I Interferon production by AMs [47], a phenomenon previously observed in activated AMs [48,49]. Congruent with a lack of involvement of cell fusion in vitro using PKH26 and CFSE-labeled cells, we did not observe changes in the expression of genes reported to be involved in such mechanisms in vivo, such as connexin 43, associated with syncytial trans-epithelial alveolar communication [50], or PanX1 and P2X7R, receptors contributing to the cell fusion mechanisms [21,51] and participating in allergic asthma pathology [52]. Intriguingly, although tAMs showed clear signs of DNA damage, we did not evidence changes in genes controlling the response to such insults, such as *Trp53*, *C-myc* and *Atr* expression. However, *Trp53* expression does not necessarily reflect a modulation of p53 functions through its phosphorylation level [53]. In line with that, we observed a decrease in the phosphorylation status of p53 in tAMs stimulated in vitro with GM-CSF, suggesting that upon GM-CSF exposure, the cells are permissive to cell division although DNA damage might be present. Similarly, ATR expression and function may be uncoupled from its mRNA expression via modulation of its phosphorylation status, in particular in the control of DNA damage associated with cell death [54] or its isomeric status [55]. In contrast, *MafB* expression increased upon in vivo HDM exposure. Interestingly, in humans, MafB immunostaining in AMs correlates with altered spirometry in smokers [56]. Further, MafB also regulates type I IFN production through recruitment of coactivators to IFN regulatory factor [57], and double-strand DNA break-induced type I IFN has been shown to enhance DNA damage response in macrophages [58]. In addition, our observation of an allergic asthma-driven increased expression of *MafB* in tAMs may be in line with reports that Mafb upregulates the expression of M2 polarization markers such as MRC1 and CD163 [59]. Nonetheless, a classical function of IL-4-induced large MNGCs (with more than 10 nuclei/cell) is the phagocytosis of large and complement-opsonized materials, which is more effective than their unfused M2 macrophage precursors [15]. Although clearance of apoptotic cells by macrophages in the airways plays a vital role in the resolution of airway inflammation [60,61], it is unclear whether polynuclear tAMs play a similar role.

Finally, epidemiologic associations between atopies and protection against cancer development have been observed [62,63]. In that context, IgE has been reported to play a key role [63], possibly due to an IgE-driven macrophage rewiring [64]. However, besides this atopic protective effect, the fact that polynuclear macrophages arise from division defect, a major mechanism in tumorigenicity, suggests that some allergic inflammation and cancer mechanisms may overlap at the molecular levels. Therefore, pharmacological targeting of allergic lungs by anti-cancer drugs may (i) provide a new approach to clarify the functions of macrophage polynucleation in the development and/or severity of allergic asthma, and (ii) might be an interesting therapeutical approach in human allergic asthma.

## 4. Material and Methods

### 4.1. Animals

Mice on a BALB/c background were bred and housed in the specific-pathogen-free animal facility of the University of Lübeck. They were handled in accordance with the appropriate institutional and national guidelines. Experimental model studies were reviewed and approved as 86-7/17 and 44-5/18, by local authorities of the Animal Care and Use Committee (Ministerium für Landwirtschaft, Energiewende, Umwelt und ländliche Räume, Kiel, Germany). Genetically deficient *Ccr2* mice and their C57Bl/6 counterpart were bred and handled in the specific-pathogen-free animal facility of the University of Cincinnati (IACUC protocol #05-01-12-02, approved 2016) in accordance with the appropriate institutional and national guidelines.

### 4.2. Antibodies and Reagents

Anti-mouse Siglec F (clone E50-2440) labeled with Brilliant Violet (BV)421 was from BD Bioscience (San Jose, CA, USA). Anti-mouse CD64 (clone X54-5/7.1) labeled with Fluorescein isothiocyanate (FITC), anti-mouse IL17RB (clone 9B10) in Phycoerythrin (PE), anti-F4/80 (clone BM8) in BV510, and unlabeled anti-mouse TSLPR (clone 22H9) were from Biolegend (San Diego, CA, USA). Anti-TSLPR antibody was stained by a secondary anti-rat F(ab)_2_ antibody labeled with Allophycocyanin (APC) (Cell Signaling Technology, Leiden, The Netherlands). Anti-mouse CD11b (clone M1/70) labeled with BV510, anti-mouse CD11c (clone N418) labeled with APC, anti-IL13Ra1 (13MOKA) in PE, anti-mouse MerTK (clone DS5MMER) labeled with PE-Cy7, anti-mouse MHCII (Clone M5/114.15.2) labelled with APC-eFluor 780, and unlabeled blocking anti-mouse CD16/CD32 antibodies were purchased by eBioscience (Thermofisher, Darmstadt, Germany). The anti-ST2 antibody (clone DJ8) labeled with APC was purchased from mdbbiosciences (Zürich, Switzerland). In addition, 4′,6-diamidino-2-phenylindole (DAPI) and FITC-labeled wheat germ agglutinin (WGA) protein were purchased from Invitrogen (Thermofisher, Darmstadt, Germany). Flow cytometry histograms show either fluorescence/counts or fluorescence/percentage of maximum (% of Max). The latter was used to provide a better comparison when the numbers of events measured in different samples differed widely. Flow cytometry data analysis were performed using FlowJo X (Flowjo, Ashland, OR, USA).

### 4.3. House Dust Mite (HDM)-Driven Allergic Asthma Model

In BALB/c mice, allergic asthma was induced by intratracheal (i.t.) application of HDM, carried out once a week for 4 consecutive weeks. BALB/c mice were anaesthetized (0.2% Xylazin & 5 mg/mL ketamine in 100 µL total) and HDM (Greer Laboratories, Lenoir, NC, USA) (100 µg/50 µL PBS) or PBS given i.t. Mice were sacrificed 72 h after the last challenge (Figure S1A). In C57Bl/6 mice (and *Ccr2^−/−^* on a C57Bl/6 background), HDM-triggered allergic asthma was established by intra-peritoneal injections of HDM (10 µg in PBS) twice on week 1 and 2, followed by intra-tracheal application of HDM (100 µg/50 µL PBS) on week 3 and 4. Lungs were harvested 72 h after the last challenge (Figure S1B).

### 4.4. Assessing the Allergic Asthma Phenotype

To measure the airway hyperresponsiveness (AHR), mice were anaesthetized and AHR measured upon mechanical ventilation using a FlexiVent (SciReq, Montreal, QC, Canada) system as described [65]. Aerosolized Acetyl-β-Methyl-Choline (methacholine) (0, 2.5, 5, 10, 25, and 50 mg/mL; Sigma-Aldrich, Steinheim, Germany) was generated by an ultrasonic nebulizer and delivered in-line through the inhalation port for 10 s. Airway resistance was measured 2 min later.

To assess the cellular infiltration in the airways, BAL fluid samples were obtained by cannulating the trachea, injecting 1 mL of ice-cold PBS, and by subsequently aspirating the BAL fluid. After red blood cell lysis, BAL fluid cells were washed once in PBS and counted using a Neubauer chamber (VWR International, Darmstadt, Germany). Frequencies of BAL fluid cells were determined by flow cytometry using the respective markers: Siglec F/Autofluorescence (macrophages), Siglec F (eosinophils), Ly6G (neutrophils), and CD3 and CD4 (T cells). Cell numbers were calculated using the cell-specific frequency of total and total cell counts/mL.

### 4.5. Lung Single Cell Suspension and Alveolar Macrophages Isolation

Lung harvests were performed as described previously [28]. Briefly, after flushing the airways with 1 mL PBS, lung lobes were taken out, placed in a 40 µm strainer, cut into small pieces, and incubated with 0.25 mg/mL liberase TL (Roche, Rotkreuz, Switzerland) and 0.5 mg/mL DNAse I (Sigma-Aldrich, Steinheim, Germany) in RPMI (Life Technology, Carlsbad, CA, USA). for 45 min. at 37 °C under gentle agitation. Then, lungs and cell suspension were pressed using a syringe plunger and washed with 10 mL wash media before centrifugation (350× *g*, 10 min.), and the excess of red blood cells present in the isolation was removed by red blood cell lysis. After washing, pulmonary cell suspension was blocked with an anti-CD16/32 antibody (eBioscience, Darmstadt, Germany) and stained. The tAM population was sorted by Fluorescence activated cell sorting (FACS) as live SiglecF^+^ CD11c^+^ cells, using an Aria III cell sorter (BD Bioscience, San Jose, CA, USA) [28]. In some cases, MHCII was added to discriminate tAM subpopulations. For simple measurements, cells were washed after staining and measured using an LSRII analyser (BD Bioscience, San Jose, CA, USA).

### 4.6. RNA Isolation and cDNA Synthesis, Real Time Semi-Quantitative Polymerase Chain Reaction

To assess the effect of HDM on the expression level of different genes by RT-PCR, freshly sorted SiglecF^+^ CD11c^+^ MHCI^−^ or MHCII^+^ tAMs were lysed using 150 µL Trizol (Invitrogen, Thermofisher, Darmstadt, Germany) and total RNA isolated using Direct-zol^TM^ RNA Mini-Prep kit (Zymo Research, Freiburg, Germany) according to the manufacturer’s instructions. Finally, freshly isolated RNAs were reverted into complementary (c)DNAs using PrimeScript RT Reagent kit, according to the manufacturer’s instructions (TAKARA Bio, Saint-Germain-en-Laye, France). Quantitative PCR was done using iQ Syber green (Bio-rad Laboratories, Münich, Germany) on a CFX96 real-time PCR system (Bio-rad Laboratories, Münich, Germany) using the specific primers (Eurofins, Ebersberg, Germany). Raw data were analyzed using CFX Maestro^TM^ Software (Bio-rad Laboratories, Münich, Germany). As a housekeeping gene, *S14* mRNA, encoding for the ribosomal protein, which is a part of the 40S ribosomal subunit, was used [66]. The primers used are described in Table 1. Finally, the relative abundance of target genes was calculated as mRNA abundance = (1.8⁽^cp^ ˢ¹⁴^−^ᶜᵖ ᵍᵉ^n^ᵉ⁾) * 100,000.

### 4.7. Primary Cell Culture for Immunofluorescence Microscopy

In order to evaluate the effect of cytokines on the ploidy of tissue-associated alveolar macrophages (tAMs), freshly sorted primary SiglecF^+^ CD11c^+^ tAMs (100–150 × 10^3^ cells) were seeded with or without GM-CSF (10 ng/mL), IL-5 (50 ng/mL) (Peprotech corporation, Rocky Hill, USA), IL-13 (50 ng/mL), IL-33 (50 ng/mL) (eBioscience Thermofisher, Darmstadt, Germany), or IL-4 (1%), for 7 days on paramount slides in RPMI complete media supplemented with Penicillin/Streptomycin, L-Glutamin (all from Life Technology, Calsbad, CA, USA) and 10% FCS (PAA, Thermofisher, Darmstadt, Germany), 37 °C, 5% CO_2_. Media was changed (including the cytokines) on day 2, 3 and 5. After 7 days, slides were washed with PBS, fixed with 2% formalin and stained with DAPI and wheat agglutinin protein (WGA, 1:100) for 15 min at RT in the dark. Finally, slides were washed to get rid of the excess of fluorochromes, covered with fluroshield and glass cover and examined using Laser Scanning Microscope FV1000 (Olympus corporation, Tokyio, Japan) (Figure S1C). For evaluation, five pictures per well were taken (four from each of the corners and one from the middle of the well) under 20× oil, 40×, 60× oil objectives. Raw data interpretation was performed using IMARIS (Bitplane, Concord, CA, USA) allowing the evaluation of the percentage of binucleated or polynucleated cells, hence giving a binucleation index. Of note, multinucleated cells were not confused with apoptotic cells (Figure S1D). To assess the activity of IL-4 in driving cell fusion, bone-marrow-derived macrophages were differentiated with *Macrophage Colony-Stimulating Factor* for 4 days before being kept for an additional 3 days in fresh medium containing 1% IL-4 conditioned medium coming from IL-4 secreting cells as described previously [15]. Furthermore, using Image J (NIH, USA), pictures were mathematically processed to calculate the area of nuclei, and the area below 5000 AU was removed from analysis after visual validation, as possible debris from dead cells. For cell-cell fusion assay, sorted SiglecF^+^ CD11c^+^ tAMs were divided into two pools, stained either with PKH26 (Sigma-Aldrich, Steinheim, Germany) or Carboxyfluorescein succinimidyl ester (CFSE) (Thermofisher, Darmstadt, Germany) according to manufacturer’s recommendations (Figure S1E). After staining, cells were mixed at a 1:1 ratio and seeded on permanox slides 100–150 × 10^3^ cells/well and cultivated with GM-CSF for 5 days with changes on day 2 and 3. Cells were counterstained with DAPI, washed and mounted before visual examination.

### 4.8. Statistical Analysis

Statistical analysis was performed using GraphPad Prism (version 8.0.1; GraphPad Software, Inc., LaJolla, USA). The graphs are presented as scatter plots with bars showing the individual samples and the mean ± standard error of the mean (SEM) respectively. Normal distribution of data was tested, when meaningful, using the Kolmogorov–Smirnov and D’Agostino–Pearson tests. Statistical differences between two groups were assessed by unpaired *t* test. Comparisons involving multiple groups were first evaluated by an analysis of variance (ANOVA) followed by Tukey’s multiple comparison test. When groups were not normally distributed, they were analyzed using a Mann–Whitney U (two groups), or ANOVA on ranks (multiple groups) followed by a Dunn’s multiple comparison test. A *p* value < 0.05 was considered statistically significant * *p* < 0.05; ** *p* < 0.01; ^§§§^ *** *p* < 0.001).

## Figures and Tables

**Table 1:** Primer list and description.

Primer Name	Sequence (5′–3′)
*mP2rx7*_F	GACAAACAAAGTCACCCGGAT
*mP2rx7*_R	CGCTCACCAAAGCAAAGCTAAT
*mPanx1*_F	CCACCGAGCCCAAGTTCAA
*mPanx1*_R	GGAGAAGCAGCTTATCTGGGT
*mTrp53*_F	CTCTCCCCCGCAAAAGAAAAA
*mTrp53* R	CGGAACATCTCGAAGCGTTTA
*mAtr*_F	GAATGGGTGAACAATACTGCTGG
*mAtr* R	TTTGGTAGCATACACTGGCGA
*mMafb*_F	TTCGACCTTCTCAAGTTCGACG
*mMafb* R	TCGAGATGGGTCTTCGGTTCA
*mGja1*_F	ACAGCGGTTGAGTCAGCTTG
*mGja1* R	GAGAGATGGGGAAGGACTTGT
*mMyc*_F	ATGCCCCTCAACGTGAACTTC
*mMyc* R	CGCAACATAGGATGGAGAGCA
*S14*_R	TGGCAGACACCAAACACATT
*S14*_F	GAGGAGTCTGGAGACGACGA

## Supplementary Materials

The following are available online at https://www.mdpi.com/1422-0067/21/20/7487/s1.

## Abbreviations

tAM	tissue-associated alveolar macrophage
BAL	bronchoalveolar lavage fluid
BiN	binucleated
BM	Bone marrow
CD	cluster of differentiation
GM-CSF	Granulocyte Macrophage-Colony Stimulating Factor
HDM	house dust mite
IFN	interferon
IL	interleukin
M-CSF	Macrophage-Colony Stimulating Factor
MFI	mean fluorescence intensity
MHC	major histocompability complex
MNGC	multinuclear giant cell
TB	tuberculosis
TSLP	Thymic stromal lymphopoietin
WGA	wheat germ agglutinin
WT	wild type
