# Supplementary material for: GM-CSF and IL-33 Orchestrate Polynucleation and Polyploidy of Resident Murine Alveolar Macrophages in a Murine Model of Allergic Asthma
Source: Int J Mol Sci. 2020 Oct 11;21(20):7487. doi: 10.3390/ijms21207487 (PMC7589978; doi:10.3390/ijms21207487)
Supplement: Supplementary file 1 [file ijms-21-07487-s001.pdf]

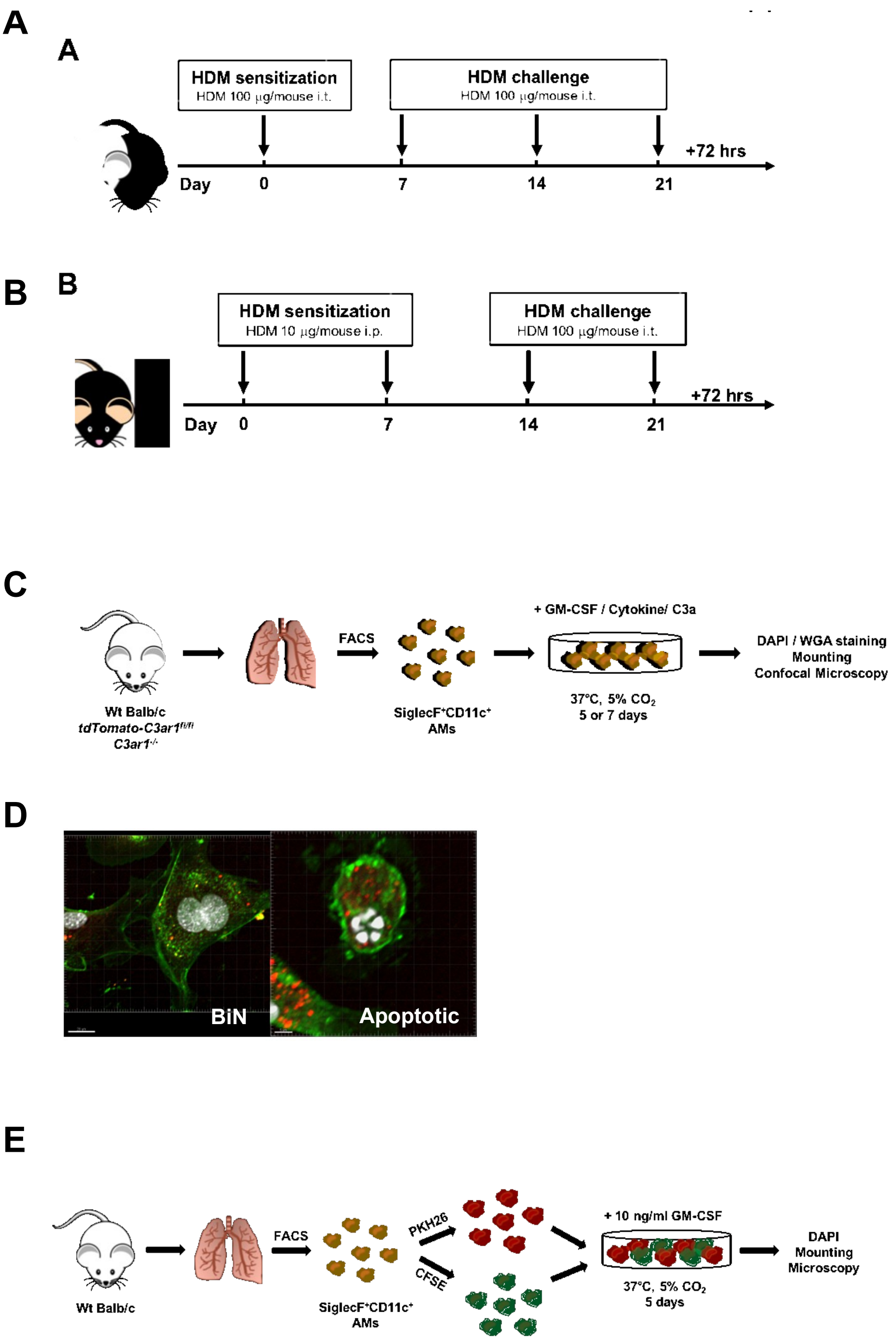

Figure S1. Models (**A**) Allergic asthma effector model was established by i.t. application carried out once a week for 4 consecutive weeks. Balb/c mice were anaesthetized and house dust mite extract (100 μg/50 μL PBS) or PBS alone was applicated intra-trachealy, and the mice were sacrificed 72 h after the last challenge. (**B**) HDM-triggered allergic asthma in C57BL/6 WT, and *Ccr2*$^{-/-}$ mice was established by intra-peritoneal injections of HDM (10 ug in PBS) twice on week 1 and 2, followed by intra-tracheal application of HDM (100 μg/50 μL PBS) on week 3 and 4. Lungs were harvested 72 h after the last challenge. (**C**) Effect of cytokines on the ploidy of tissue-associated alveolar macrophages (tAMs); freshly sorted primary tAMs (100–150 × 10^3 cells) were seeded with or without GM-CSF (10 ng/mL), IL-13 (50 ng/mL), IL-4 (1%), IL-5 (50 ng/mL), or IL-33 (50 ng/mL) for 7 days before fixation and staining with DAPI and wheat agglutinin protein. Each condition was examined by fluorescence microscopy. (**D**) Visual examination of cells after DAPI/WGA staining allows the

identification of Binucleated (BiN) cells that were not confused with apoptotic cells. Pictures were taken using a x60 oil-immersion objective (**E**). Cell-cell fusion assay; sorted tAMs were divided into two pools, stained either with PKH26 or CFSE, then were mixed with a 1:1 ratio, seeded on permanox slides 100–150 × 10^3 cells/well and cultivated with GM-CSF for 5 days. Cells were counterstained with DAPI washed and mounted before visual examination.

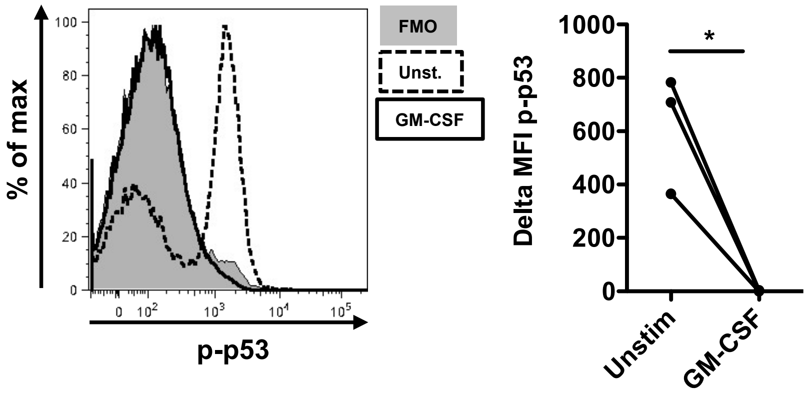

Figure S2. GM-CSF decreases the phosphorylation status of p53 in sorted tissue-associated alveolar macrophages. Tissue-associated alveolar macrophages (tAMs) from naive mice were sorted based on CD11c and Siglec F expression and kept in culture in complete RPMI medium with 10% FCS for 3 days in the presence or absence of 10 ng/mL GM-CSF. After stimulation, the tAMs were detached and harvested using TEN buffer (Tris Base 40 mM, EDTA 1 mM, NaCl 150 mM, pH 8.0), fixed with 4% paraformaldehyde and permeabilized with 0.2% Tween in PBS. After blocking with Fc block in PBS/BSA, cells were stained for 30 min using a mouse anti-phospho-p53 (Ser 15) (Clone 16G8, Cell Signaling Technology) followed by a secondary staining using a donkey anti-mouse labeled with Alexa-488 (Abcam). Histogram overlays are representative of n = 3 isolations and show the FMO (grey histogram) and the p-p53 signal in unstimulated (dotted line) and GM-CSF (plain line) treated cells. Data show the mean value of the MFI normalized to FMO control (Delta MFI) ± SEM from n = 3 isolations. Statistical significance was assessed using a paired t-test. * $p < 0.05$.
